# Unveiling a Hidden Pathogen: The Role of Sphingomonas paucimobilis Beyond the Hospital Walls

**DOI:** 10.7759/cureus.84414

**Published:** 2025-05-19

**Authors:** Sharon Nguyen, Maryam Naushab, Lavanya Srinivasan

**Affiliations:** 1 Internal Medicine, Baylor Scott and White All Saints Medical Center, Fort Worth, USA

**Keywords:** dental abscess, dental cavity, hemopytsis, lung abscess, sphingomonas paucimobilis, tooth infection

## Abstract

Dental abscesses or cavities can lead to the formation of cavitary lung lesions through aspiration or by initially entering the bloodstream. The most common causative organisms are typically gram-negative or anaerobic species. *Sphingomonas paucimobilis* is a widespread organism, but it is generally regarded as an uncommon pathogen, primarily linked to nosocomial infections or community-acquired infections. We report a potential novel link between *Sphingomonas paucimobilis* and dental abscesses. Research on this particular bacterium is currently limited, leaving gaps in our understanding of its characteristics, behavior, and potential treatment options. As a result, the recognition of this organism by healthcare providers is essential for early identification and effective management.

## Introduction

*Sphingomonas paucimobilis* is an aerobic, gram-negative bacillus that is present in both environmental and hospital settings. It is reported as a causative organism in many nosocomial infections such as peritoneal dialysis catheters, hemodialysis devices, indwelling catheters, and ventilators [1]. *Sphingomonas paucimobilis* has also been implicated in bone and soft tissue infections such as osteomyelitis, cellulitis, and septic arthritis [2]. Dental abscess typically arises from untreated dental caries, trauma, or complications from previous dental procedures [2,3]. Spread of these untreated dental infections poses the risk of seeding into the neck or lung, which can potentially result in Ludwig angina, mediastinitis, necrotizing fasciitis, involvement of the retropharyngeal space, and lung cavitary lesions [4,5]. Although *S. paucimobilis* is not typically implicated in dental abscess, there have been a few reported cases of this pathogen linked to an odontogenic source such as gingival ulcer or retropharyngeal abscess in the setting of poor dentition with a lack of proper dental care [6-9]. We report an unusual presentation of *S. paucimobilis* bacteremia in a 21-year-old male without any significant comorbidities presenting with tooth infection, hemoptysis, and right upper lobe cavitary lesion. His clinical presentation was further complicated by a six-month history of incarceration. The patient received empiric antibiotics and underwent bronchoscopy with lavage, which showed chronic inflammation without significant microorganism growth. This is the first known case of cavitary lung lesions associated with *S. paucimobilis* bacteremia, complicated by a concurrent tooth infection.

## Case presentation

A 21-year-old Hispanic male with a past medical history of asthma presented to the emergency department with a new onset of fever with episodes of intermittent hemoptysis. The patient described the hemoptysis to be bright red without any blood clots, a teaspoon amount, occasionally mixed with mucus, and often occurring following multiple coughing episodes. He was unable to correlate with any alleviating or exacerbating factors. The patient stated that he had not tried any medications at home prior to hospitalization for symptom control. He also had never experienced these symptoms before. At the time of examination, he reported subjective fevers at home without any myalgia or generalized weakness. Otherwise, he denied any night sweats or unintentional weight loss. He also denied any chest pain, shortness of breath, or dyspnea on exertion. He reported no concerns for gastrointestinal bleeding symptoms including hematochezia, melena, or hematemesis. He also denied any worsening dental or facial pain in the last few months. Of note, the patient stated that he was incarcerated for a total of six months this past year. He was able to recall two negative QuantiFERON gold tuberculosis tests in May 2024 prior to his incarceration period and again after his release date in November 2024. He also mentioned a chronic history of untreated dental cavities prior to being incarcerated. He denied any history of dental procedures or recent antibiotic treatment, as well as any current or past recreational drug use, daily alcohol use, or tobacco use. He was unemployed and lived at home with his family, who were all reportedly healthy. His only reported medication was an albuterol inhaler.

Initial vitals were as follows: temperature of 100°Fahrenheit, heart rate of 106 beats per minute, respiration of 18 breaths per minute, blood pressure of 116/76 mm Hg, and oxygenation saturation of 99% on room air. Initial and pertinent labs are further outlined in Table 1. Physical examination of the oral cavity showed slight yellow discoloration of teeth with no loose, broken, or chipped teeth. Gums were slightly erythematous without any bleeding or swelling appreciated. Oral mucosa appeared moist, pink without any discoloration, lesions, nodules, or swelling. Tonsils were visible but not enlarged. Other pertinent physical examination findings including cervical, cardiac, and pulmonary were unremarkable.

**Table 1:** Lab investigation NAA, nucleic acid amplification

Lab Investigation	Patient Value	Reference Values
Comprehensive metabolic panel		
Sodium	136 meq/L	136-145 meq/L
Potassium	3.8 meq/L	3.6-5.0 meq/L
Chloride	104 meq/L	98-107 meq/L
Carbon dioxide	28 meq/L	21-32 meq/L
Blood urea nitrogen	10 mg/dL	7-18 mg/dL
Creatinine	1.02 mg/dL	0.70-1.30 mg/dL
Glucose	85 mg/dL	70-99 mg/dL
Complete blood count		
White blood cell count	18.0 10^3^/uL	4.5 -11.0 10^3^/uL
Red blood cell count	4.72 10^6^/uL	4.50-6.00 10^6^/uL
Hemoglobin	13.5 g/dL	14.0-18.0 g/dL
Hematocrit	42.0%	42.0-52.0%
Mean corpuscular volume	89.0 fL	88.0-99.0 fL
Platelets	446 10^3^/uL	150-450 10^3^/uL
Inflammatory markers		
Lactic acid	0.9 mmol/L	0.9-1.7 mmol/L
Erythrocyte sedimentation rate	44 mm/h	0-15 mm/h
C-reactive protein	10.6 mg/dL	0.0-0.03 mg/dL
Respiratory viral panel		
SARS-CoV-2 (COVID-19) virus, NAA	Not detected	Not detected
Influenza A, NAA	Not detected	Not detected
Influenza B, NAA	Not detected	Not detected
Group A Streptococcus	Negative	Negative

**Figure 1:**
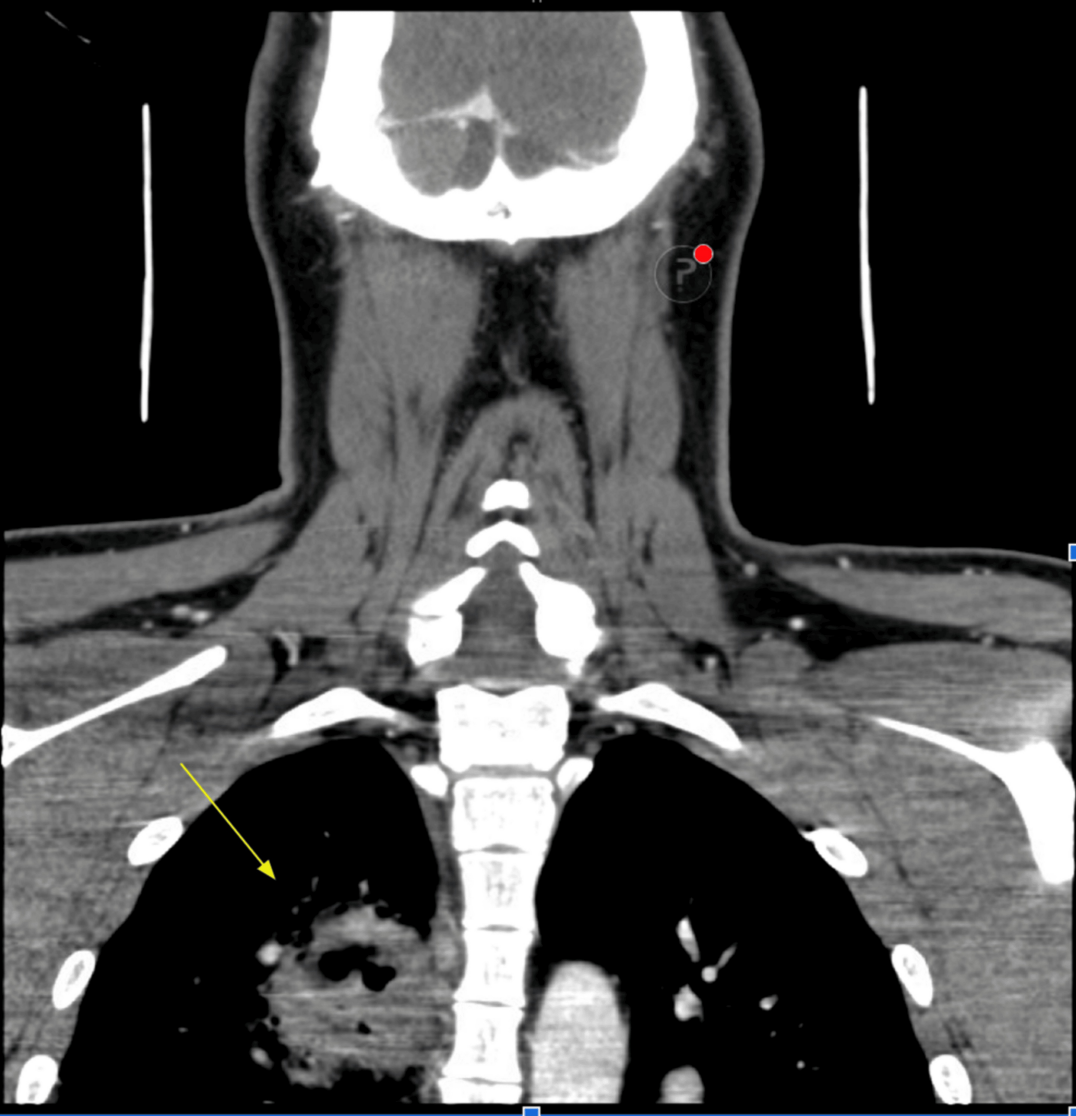
CT scan of the soft tissue neck with contrast CT scan of the soft tissue neck with contrast showing a thick-walled cavitary lesion measuring approximately 6 x 5.5 x 4.4 cm in the posterior inferior aspect of the right upper lobe. Cavitary is air-filled. No definite communication with the bronchi is identified. CT, computed tomography

**Figure 2:**
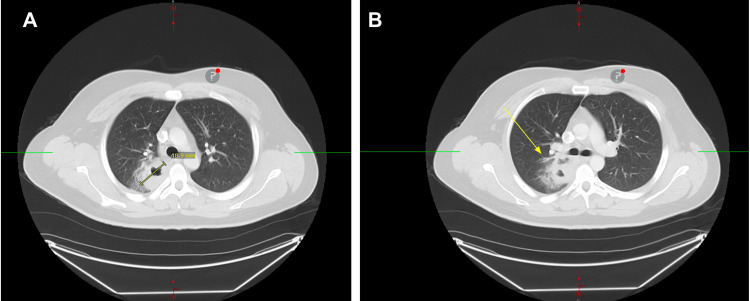
CT of the chest with contrast (A, B) CT chest with contrast performed showing a thick-walled irregular cavitary lesion in the posterior medial right upper lobe posterior to the right hilum measuring approximately 4.6 x 4.8 x 4.5 cm. The right hilar lymph nodes are mildly enlarged, and there is adjacent inflammatory change. Lungs are otherwise clear without any pleural effusion or pneumothorax. CT, computed tomography

**Figure 3:**
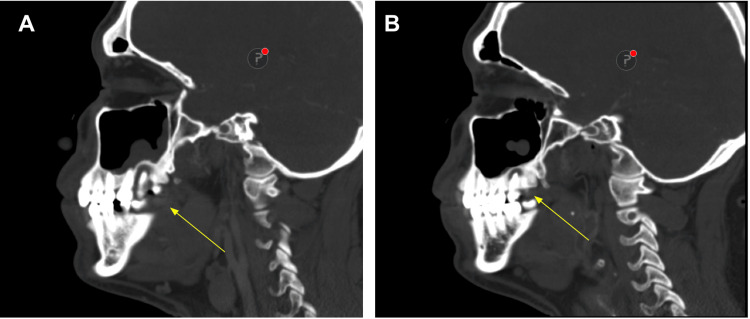
CT of the maxillofacial area with and without IV contrast CT of the maxillofacial area with and without IV contrast showing (A) right side of the face and (B) left side of the face. Cavities and periapical abscesses are noted involving the maxillary and mandibular teeth, more prominent on the right side than the left side. Mucosal thickening of the biliary maxillary sinus is seen on the right side more than the left side. CT, computed tomography

The patient underwent a series of radiological imaging consisting of computed tomography (CT) of the maxillofacial area, neck, and chest. CT of the maxillofacial area showed periapical abscesses and cavities involving the maxillary and mandibular teeth and right-sided facial cellulitis without any discrete soft tissue abscesses amenable to drainage. CT of the neck and chest demonstrated a fairly large cavitary lesion in the posterior medial right upper lobe near the hilum (Figures 1-3). Based on the patient’s history, physical examination, and lab and radiological findings, tuberculosis, actinomycosis, aspergillosis, *Nocardia*, and malignancy were on our differentials.

The collection of expectorated sputum was obtained on three consecutive days and sent for acid-fast bacilli smear and culture. By day 2 of hospitalization, the patient’s blood cultures interestingly grew *S. paucimobilis*. Additional infectious work-up including acid-fast bacilli from sputum, fungal antibody, and other pertinent labs is outlined in Table 2.

**Table 2:** Infectious work-up *MRSA PCR nares: this is a test used to detect the presence of MRSA bacteria in the nasal passages. **Acid-fast bacilli culture x 3: sputum was obtained on three consecutive days and sent for acid-fast bacilli smear, and all three results were negative. MRSA PCR, methicillin-resistant *Staphylococcus aureus* polymerase chain reaction

Lab Investigation		
Blood cultures x 2	2 out of 2 blood cultures positive: *Sphingomonas paucimobilis*	
Histoplasma antibody	Negative	Negative
Blastomyces antibody	Negative	Negative
Coccidioides antibody	Negative	Negative
Aspergillus antibody	Negative	Negative
MRSA PCR nares*	Not detected	Not detected
Cryptococcal antigen	Negative	Negative
Acid-fast bacilli culture x 3**	No acid-fast bacilli isolated	Negative, positive if acid-fast bacilli isolated
*Nocardia* culture, from sputum	No filamentous bacteria seen	Negative, positive if *Nocardia* species isolated

The patient was started on broad-spectrum antibiotic treatment with intravenous piperacillin-tazobactam on the first day of hospital admission and continued following the bronchoscopy evaluation. The bronchoscopy was performed after three negative acid-fast stain results. Bronchoscopy evaluation yielded evidence of mild inflammation around the right upper lobe posterior segment, but no other obvious endobronchial lesion was visualized. Cytology of the bronchial alveolar lavage, washings, and brushing at the right upper lobe demonstrated reactive bronchial epithelial cells in the background of mucus and mixed acute and chronic inflammation. Negative malignant cells were reported. Additional cultures to evaluate for *Nocardia*, acid-fast bacteria, fungal, and mycobacterium tuberculosis at both the bronchus and right upper lobe were all unremarkable. Repeat blood cultures were collected on day 4 of antibiotic treatment, which again showed no growth for at least 48 hours. Of note, oral surgery or dental evaluation was recommended; however, no specialty coverage was available for inpatient consultation. The patient continued to receive empiric antibiotics during hospital stay, with clinical improvement in hemoptysis and fevers. The patient was then discharged home with an antibiotic course of Augmentin indefinitely until dental evaluation, followed by pulmonary care for close monitoring of the right upper cavitary lesion.

## Discussion

*Sphingomonas paucimobilis* is often associated with primary bacteremia, commonly presenting as pneumonia or catheter-related infections, particularly in individuals with compromised immune systems or other underlying comorbidities, such as diabetes, alcoholism, kidney disease requiring hemodialysis or peritoneal dialysis, or cancer patients [1,2]. In one case report, a patient with peritoneal dialysis developed relapsing peritonitis secondary to *S. paucimobilis* from 1 peritoneal dialysis catheter [6]. This patient’s treatment involved catheter removal, prolonged intravenous antibiotics with meropenem, and transition to hemodialysis thereafter [6]. This is in contrast to our case in which the patient did not have any known comorbidities that would put him at an increased risk for acquiring this infection. Therefore, this case is particularly noteworthy as *S. paucimobilis *is generally seen in patients with comorbidities or immunosuppression. This patient’s presentation suggested that the bacterium can still cause infections in otherwise healthy individuals, warranting further exploration into its potential for causing disease in a broader range of patients.

Secondly, dental abscesses that are left untreated or inadequately managed can lead to serious complications including the development of lung cavitary lesions. The bacteria responsible for dental infections, such as *Streptococcus* and *Staphylococcus *species, can spread from the site of the abscess through the bloodstream (bacteremia) or by direct extension through surrounding tissues [7]. In some cases, the infection may reach the lungs either via aspiration of infected material or through the bloodstream, resulting in the formation of lung cavitary lesions. Currently, there has been only one documented case of *S. paucimobilis* stemming from a gingival ulcer with bone exposure affecting the attached gingiva in the anterior maxillary region [8]. A case study reported that non-classical pathogens of the oral cavity, such as *S. paucimobilis*, may be the causative microorganism behind the ulcer [8]. Another similar case report mentioned *S. paucimobilis* bacteremia secondary to a retropharyngeal abscess in a patient with poor dentition, and surgical drainage was deferred due to rapid clinical improvement and decrease in size with antibiotics [9].

At this time, the definitive source of bacteremia remains unclear; however, we suspect that *S. paucimobilis *most likely originated from the periapical abscesses and dental caries noted on CT of the maxillofacial area. We found no other documented case reports clearly documenting the association between *S. paucimobilis* and dental cavity or abscess itself during literature review. Thus, this case study emphasizes a potential novel association between *S. paucimobilis* and the oral cavity that has not yet been clearly established or well-studied.

While *S. paucimobilis* infections are considered rare and primarily occur in hospital settings, there are currently no specific data regarding their prevalence or impact within incarcerated populations. People who are incarcerated often face unique challenges when it comes to health including a higher risk of being immunocompromised and having chronic conditions such as HIV, diabetes, and hepatitis that are frequently underdiagnosed or poorly managed. In addition to this, factors such as overcrowding and shared facilities pose the risk of spreading infection rapidly.

A notable limitation of this case report is the lack of available dental abscess cultures, which would then strengthen our clinical suspicion of hematogenous spread secondary to an odontogenic source. Additionally, there was no oral or dental specialty coverage available for a formal inpatient consult. To further strength the case for hematogenous spread of this pathogen, investigation for endocarditis could have been performed to rule out cardiac etiologies. However, we had low clinical suspicion for endocarditis given no positive risk factors or comorbidities. Factors such as the patient's immune status, the virulence of the infecting bacteria, and the promptness of treatment significantly influence the likelihood of such complications. Routine dental care and treatment of dental cavities are essential in preventing not only dental abscesses but also spread of infection to the lungs and the subsequent development of potentially life-threatening cavitary lesions.

## Conclusions

This case report highlights a potential association between *S. paucimobilis *and dental abscesses, which differs from what is commonly reported in the existing literature. Most studies and research to date have linked *S. paucimobilis* primarily to nosocomial infections, particularly in immunocompromised patients or those with underlying medical conditions. However, this case challenges that perspective by suggesting that *S. paucimobilis* could also play a role in the development of dental abscesses. This observation opens the door to further investigation into the potential for *S. paucimobilis* to contribute to infections outside of hospital settings, especially in relation to dental health. Potential areas for further investigation include virulence factors or this pathogen’s interaction with the oral microbiome.
